# Gender Differences in Cardiac Chronotropic Control: Implications for Heart Rate Variability Research

**DOI:** 10.1007/s10484-021-09528-w

**Published:** 2021-11-24

**Authors:** DeWayne P. Williams, Nicholas Joseph, Gina M. Gerardo, LaBarron K. Hill, Julian Koenig, Julian F. Thayer

**Affiliations:** 1 Department of Psychological Science, University of California, 4201 Social and Behavioral Sciences Gateway, Irvine, CA 92697 USA; 2 Department of Psychology, The Ohio State University, Columbus, OH USA; 3 Department of Psychiatry and Behavioral Sciences, Duke University Medical Center, Durham, NC USA; 4 Faculty of Medicine and University Hospital Cologne, Department of Child and Adolescent Psychiatry, Psychosomatics and Psychotherapy, University of Cologne, Cologne, Germany

**Keywords:** Heart period, Heart rate variability, Heart rate, Gender differences, Sex differences

## Abstract

There is a continuing debate concerning “adjustments” to heart period variability [i.e., heart rate variability (HRV)] for the heart period [i.e., increases inter-beat-intervals (IBI)]. To date, such arguments have not seriously considered the impact a demographic variable, such as gender, can have on the association between HRV and the heart period. A prior meta-analysis showed women to have greater HRV compared to men despite having shorter IBI and higher heart rate (HR). Thus, it is plausible that men and women differ in the association between HRV and HR/IBI. Thus, the present study investigates the potential moderating effect of gender on the association between HRV and indices of cardiac chronotropy, including both HR and IBI. Data from 633 participants (339 women) were available for analysis. Cardiac measures were assessed during a 5-min baseline-resting period. HRV measures included the standard deviation of inter-beat-intervals, root mean square of successive differences, and autoregressive high frequency power. Moderation analyses showed gender significantly moderated the association between all HRV variables and both HR and IBI (each *p* < 0.05). However, results were not consistent when using recently recommended HRV variables “adjusted” for IBI. Overall, the current investigation provides data illustrating a differential association between HRV and the heart period based on gender. Substantial neurophysiological evidence support the current findings; women show greater sensitivity to acetylcholine compared to men. If women show greater sensitivity to acetylcholine, and acetylcholine increases HRV and the heart period, then the association between HRV and the heart period indeed should be stronger in women compared to men. Taken together, these data suggest that routine “adjustments” to HRV for the heart period are unjustified and problematic at best. As it relates to the application of future HRV research, it is imperative that researchers continue to consider the potential impact of gender.

## Introduction

Over the previous few decades, research has found the utility of heart period variability, also referred to as heart rate variability (HRV), to be extremely beneficial in understanding the complex interplay between psychological processes and cardiovascular activity (Holzman & Bridgett, 2017; Thayer et al., 2012). Specifically, HRV is often utilized as valid and reliable measures of parasympathetic nervous system (vagal) activity. Importantly, a well-known association exists between the heart period, or inter-beat-intervals (IBI), and HRV such that lower HRV is associated with faster IBI. Vagal stimulation releases the cholinergic neurotransmitter acetylcholine (ACh), which slows spontaneous depolarization via increased potassium conductance of the pacemaker cells in the sinoatrial node (Bartos et al., 2015). Subsequently, these ACh-gated potassium channels increase indices of vagally mediated HRV and IBI (Sakmann et al., 1983). Thus, through ACh, indices of cardiac chronotropy (IBI and HR) and HRV are “hardwired” as it were.

For this reason, many researchers have both questioned and debated the validity of HRV measures as indices of purely vagal tone (e.g., de Geus et al., 2019). Recent recommendations propose researchers should calculate the coefficient of variation (cv | dividing variability by the mean) of HRV (de Geus, et al., 2019). By doing so, it is thought that the impact of the heart period on HRV is removed, evinced by the subsequent decrease in the association between adjusted (cv) HRV and measures of cardiac chronotropy (IBI and HR). However, researchers have largely ignored the potential impact demographic variables, such as gender, could have on the association between HRV and HR. In this regard, a recent a meta-analysis (172 studies, 63,612 individuals) showed women to have greater vagally-mediated HRV *and* higher HR (and lower IBI) compared to men (Koenig & Thayer, 2016). These findings represent paradoxical cardiovascular activity, as higher HRV is typically associated with longer IBI and lower HR. Therefore, it is plausible that the association between HRV and HR/IBI may differ significantly between men and women.

### The Present Study

**Fig. 1:**
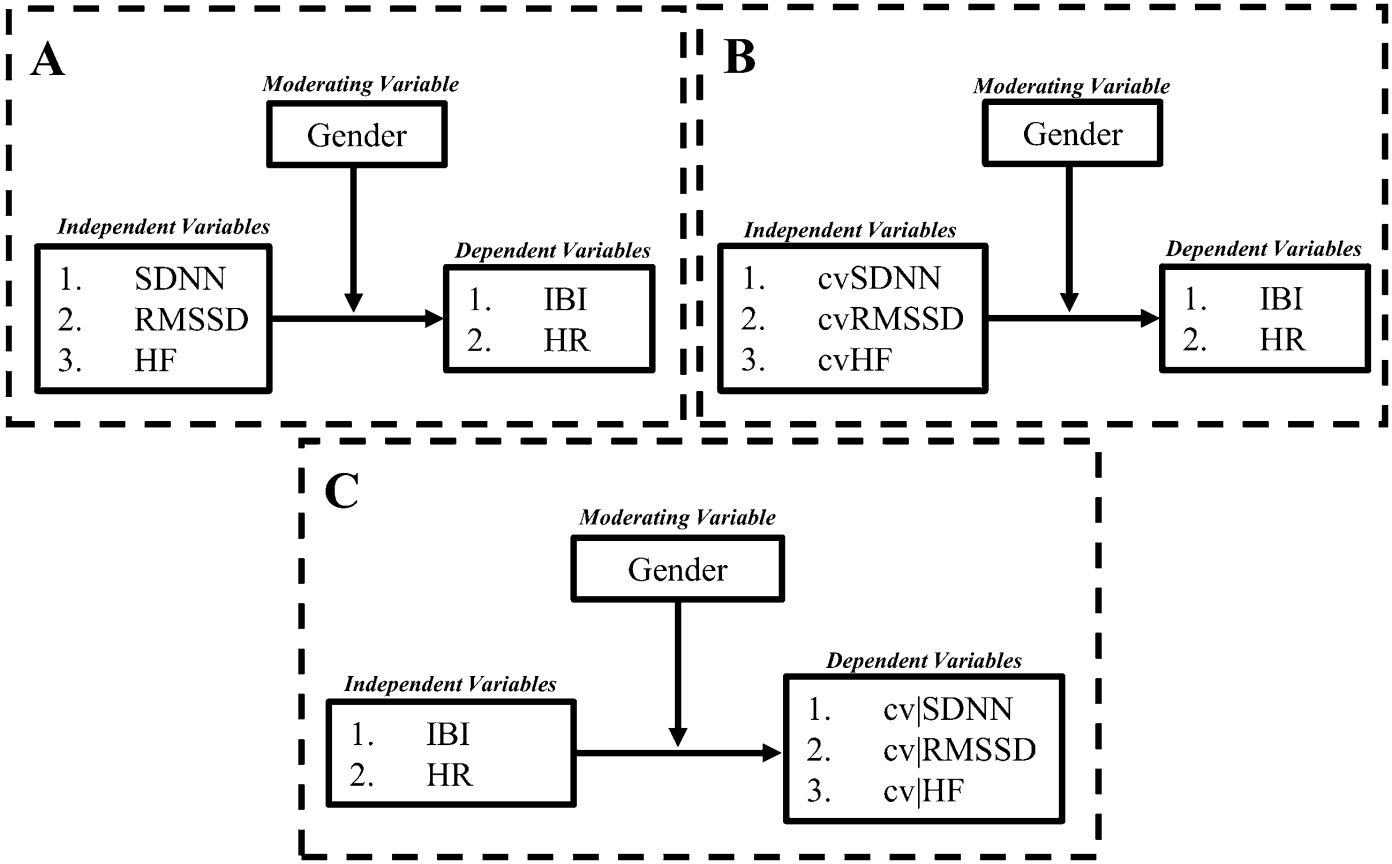
This figure represents conceptual moderation models tested in the current investigation. **A** Represents a moderation test in which measures of HRV are independent variables, gender is the moderating variable, and measures of cardiac chronotropy are dependent variables. **B** Represents a similar model, however cvHRV variables take the place of unadjusted HRV variables as independent variables. **C** Represents the reverse model such that cardiac chronotropic measures are the independent variable, gender as a moderator, and both adjusted and unadjusted measures of HRV as dependent variables

Considering that recommended cv adjustments to HRV do not account for potential gender differences (de Geus et al., 2019), the present study investigates the potential moderating effect of gender on the association between HRV (independent variables) and both IBI and HR (dependent variables) (see Fig. 1A for conceptual model). We also sought to evaluate how gender may differentially impact the association between such “adjusted” HRV parameters (de Geus et al., 2019) and the heart period as indexed by IBI and HR (see Fig. 1B for details). Along these lines, researchers also propose a unidirectional impact of cardiac chronotropy on HRV, such that the heart period impacts HRV. Thus, the following study also evaluated the reverse moderation model; that is, whether gender moderated the association between both IBI and HR (independent variables) and both unadjusted and adjusted HRV variables (dependent variables; see Fig. 1C for conceptual model). If gender variations in cardiac chronotropy does *not* predict HRV (i.e., moderation is not significant according to Fig. 1C), then it is highly unlikely that the heart period impacts HRV as some have proposed previously (de Geus et al., 2019).

## Methods

Individuals between the ages of 18 and 30 at a large midwestern institution were eligible to participate. Participants were recruited via both an introductory level psychology course research pool for partial class credit and cash compensation for participation outside of the research pool. Data were pooled across nine studies conducted within our lab, with 628 relatively young and apparently healthy individuals available for analysis. There were 338 women (mean age = 19.22 ± 1.68 years) and 290 men (mean age = 19.68 ± 2.19; total sample mean age = 19.44 ± 2.19). We asked all participants not to smoke, undergo vigorous physical activity, or drink caffeine during the 6 h prior to the experiment. Each study was approved by the institutional review board, and all participants signed written informed consent. Height, weight, gender, and age were collected prior to the start of the experiment. Participants then completed a 5-min baseline period, in which they sat in a resting position (spontaneous breathing) with a television displaying a blank, grey screen, and were instructed not to move or fall asleep.

### Cardiac Measures

Cardiac data was recorded continuously throughout each experiment via a 3-lead ECG at a 1,000 Hz sampling rate using a Mindware™ 2000D (MW2000D) Impedance Cardiograph package. Electrodes were placed (1) below the right clavicle, (2) on the left side of the abdomen (below the heart), and (3) on the right side of the abdomen. Participants' successive IBIs (in milliseconds) were extracted using Mindware™ HRV Analysis software from the electrocardiogram trace. IBIs were written in a text file and analyzed using Kubios HRV analysis package 2.0 (Tarvainen et al., 2014), allowing for the calculation of mean HR, IBI, and both time- and frequency-domain indices of resting HRV. Artifacts within the R-to-R series were visually detected, and we applied an artifact correction level that would differentiate and remove artifacts (differing abnormal IBIs from the mean IBI; see Tarvainen et al., 2014, for review) using a piecewise cubic spline interpolation method. Smoothness priors was used as a detrending method. The standard deviation of IBI (SDNN) was calculated and is considered an index of HRV that reflects both vagal (primarily) and sympathetic influence. The root mean square of successive differences (RMSSD), measured in milliseconds, was calculated as the time-domain measure of vagally mediated HRV. Autoregressive estimates were also calculated, yielding high-frequency (HF; 0.15–0.4 Hz) power HRV. Additionally, high-frequency peak values (HF_peak_) were obtained from the autoregressive spectrum as a measure of respiration rate to control for potential bias (Thayer et al., 2002). HF and LF (0.04–0.15 Hz) in normalized units (HF_n.u._ and LF_n.u._) were also derived from Kubios. The LF/HF ratio was also derived as an index of LF relative to HF power. Finally, we applied a recommended coefficient of variation (cv) “adjustment” formulae to SDNN, RMSSD, and HF yielding cvSDNN (SDNN ÷ IBI), cvRMSSD (RMSSD ÷ IBI), and cvHF (HF ÷ IBI^2^) respectively (de Geus et al., 2019).

In sum, measures of cardiac chronotropy included HR and IBI, and HRV included SDNN, RMSSD, and HF. In addition, cvHRV (adjusted HRV) variables included cvSDNN, cvRMSSD, and cvHF. Significantly skewed variables, including HR, IBI, SDNN, HF, RMSSD, cvSDNN cvRMSSD and cvHF were natural log transformed (ln) to fit assumptions of linear analyses.

### Statistical Analyses

All statistical tests were conducted using SPSS (ver. 20, IBM Chicago, IL, USA). All tests were two-tailed, and significance levels were evaluated using an alpha of 0.05.

We first stratified subjects into groups based on their gender (women and men). Participants self-reported their gender (woman or man), and experimenters independently recorded their gender (woman or man); in all subjects, the experimenters’ and subjects’ reported gender was consistent (i.e., there were no inconsistencies between reported gender). Independent samples t-tests were conducted to explore potential differences between women and men on all variables. T-values, Cohen’s *d*, and *p*-values are reported. 95% confidence intervals for Cohen’s *d* on all mean differences are reported in Table 1.

**Table 1:** Mean differences between women and men on all variables

	(A) all Individuals		(B) Women		(C) Men		*t*	*p*	*d*	Lower	Upper
	M	SD	M	SD	M	SD					
BMI	23.93	5.09	23.31	5.49	24.66	4.48	3.33	**0.001**	0.267	0.110	0.425
Respiration	0.25	0.06	0.26	0.06	0.23	0.06	− 4.30	**<** ***0.001***	− 0.500	− 0.341	− 0.659
HR	73.95	10.64	75.39	11.06	72.27	9.89	− 3.69	**<** ***0.001***	− 0.296	− 0.454	− 0.138
IBI	834.77	123.75	819.40	127.37	852.69	117.07	3.39	**0.001**	0.271	0.113	0.429
SDNN	52.49	20.24	49.78	19.13	55.64	21.05	3.65	**<** ***0.001***	0.292	0.135	0.450
(ln)SDNN	3.89	0.38	3.84	0.38	3.95	0.38	3.74	**<** ***0.001***	0.289	0.132	0.447
cvSDNN	6.24	2.10	6.02	1.95	6.50	2.24	2.85	**0.005**	0.230	0.072	0.387
RMSSD	50.21	26.07	48.84	24.93	51.81	27.29	1.43	0.154	0.114	− 0.043	0.271
(ln)RMSSD	3.79	0.50	3.77	0.49	3.82	0.50	1.34	0.179	0.101	− 0.056	0.258
cvRMSSD	5.87	2.62	5.79	2.37	5.97	2.89	0.84	0.399	0.069	− 0.088	0.226
HF	1327.38	1451.32	1347.49	1433.58	1303.95	1473.87	− 0.37	0.708	− 0.030	− 0.187	0.127
(ln)HF	6.72	0.99	6.74	0.99	6.70	1.00	− 0.47	0.637	− 0.040	− 0.197	0.117
cvHF	0.176	0.177	0.184	0.176	0.168	0.178	− 1.13	0.258	− 0.090	− 0.066	0.247
LF (n.u)	57.88	18.63	53.14	18.25	63.40	17.55	7.15	**<** ***0.001***	0.572	0.412	0.732
HF (n.u)	42.05	18.63	46.76	18.28	36.55	17.53	− 7.11	**<** ***0.001***	− 0.569	− 0.409	− 0.729
LF/HF	2.10	2.15	1.62	1.53	2.65	2.60	6.14	**<** ***0.001***	0.492	0.333	0.651

Bold-italic values denote statistical significance (*p* < .05)

This Table presents the means and standard deviations (in brackets) on variables of interest both in the full sample (A) and stratified by women (B) and men (C). Independent samples t-test statistics include both *t-* and *p*-values on the difference between men and women, in addition to Cohen’s *d* and associated 95% confidence intervals (significant *p*-values bolded). Body mass index (BMI) was measured in kg/m^2^; respiration is indexed using HF peak values; HR (heart rate) is in beats per minute; IBI (inter-beat-intervals) are in milliseconds; SDNN represents the standard deviation of inter-beat-intervals (ms); RMSSD reflects the root mean square of successive differences (ms); HF represents high-frequency power (ms2); LF represents low-frequency power; LF/HF reflects the low-to-high frequency ratio; (ln) represents natural log-transformed values; cv represents coefficient of variation

Zero-order correlations (Pearson’s *r)* were used to assess the relationship between previously indicated measures of HRV and cardiac chronotropy. Pearson’s r correlation coefficients and *p* values are reported. Correlation tests were also split by gender to compare correlation coefficient differences between men and women using Fisher’s r-to-z transformation (Steiger, 1980).

In order to test if gender moderated the relationship between HRV and cardiac chronotropy, the SPSS macro PROCESS was used (Hayes, 2012). In the program PROCESS, “Model 1” was used to test the interactive effect of HRV measures (independent variable) and gender (moderator; 1 = man, 2 = woman) on cardiac chronotropy (IBI and HR; dependent variables; see Fig. 1A). We also tested if gender moderated the association between cvHRV variables and both IBI and HR (see Fig. 1B). Finally, we tested the reverse of these models, that is, IBI and HR as independent variables and HRV measures (both adjusted and unadjusted) as dependent variables. Conditional effects were used to determine the differential relationship between men and women on the association between HRV measures and cardiac chronotropic measures using simple slope analyses. Johnson-Neyman region of significance tests were used to determine how men and women differ in cardiac chronotropy at low, mean, and high levels of HRV. High and low values for the predictor variables are derived using ± 1SD from the mean. Statistics reported include unstandardized beta (B) coefficients, standard errors (SE; in brackets), 95% Bootstrapping confidence intervals (in square brackets, 5000 samples; Hayes, 2012), partial correlation coefficients (for main effects and interactions), and *p* values. Natural log-transformed cardiac chronotropy (both IBI and HR) and HRV measures (both unadjusted and adjusted) were used in zero-order correlation and moderation tests to fit assumptions of linear analyses as these measures were significantly skewed.

## Results

### Sample Characteristics

Table 1A shows mean and standard deviation values for all variables. Table 1B and C present these values separately in women and men, respectively. Women had lower BMI (t _(626)_ = 3.33, *p* = 0.001, *d* = 0.267) and higher respiration (t _(626)_ = − 4.30, *p* < 0.001, *d* = 0.500) compared to men.

Women exhibited higher HR (t _(626)_ = − 3.69, *p* < 0.001, *d* = 0.296) and HF_n.u._ (t _(626)_ = − 7.11, *p* < 0.001, *d* = 0.596) compared to men. Women also had lower IBI (t _(626)_ = 3.39, *p* = 0.001, *d* = 0.271), SDNN (t _(626)_ = 3.65, *p* < 0.001, *d* = 0.292), natural log-transformed SDNN (t _(626)_ = 3.74, *p* < 0.001, *d* = 0.289), cvSDNN (t _(626)_ = 2.85, *p* = 0.005, *d* = 0.230), LF_n.u._ (t _(626)_ = 7.15, *p* < 0.001, *d* = 0.572), and LF/HF ratio (t _(626)_ = 6.14, *p* < 0.001, *d* = 0.492), compared to men. Notably, although not statistically reliable, women trended to have lower RMSSD but higher HF power across calculation types (see Table 1 for details). These results are consistent with meta-analytic findings (Koenig & Thayer, 2016).

### Zero-Order Correlations Between Variables of Interest

Correlation coefficients between variables of interest are presented in Table 2A for the full sample. In all individuals, there was a near-perfect negative association between IBI and HR (r = − 0.999, *p* < 0.001). Higher IBI and lower HR were associated with higher SDNN, cvSDNN, RMSSD, cvRMSSD, HF, and cvHF (each *p* < 0.001). It is important to note the correlation coefficients between cvHRV measures (e.g., cvRMSSD) and both IBI and HR are significantly smaller than associations with unadjusted HRV variables (each *p* < 0.05). This suggests significant shared variance between measures of HRV and cardiac chronotropy.

**Table 2:** Correlation coefficients stratified by gender

A: Full sample	1	2	3	4	5	6	7	8	9
1. HFhz	–								
2. HR	− .075	–							
3. IBI	.069	− .999**	–						
4. lnSDNN	− .158**	− .518**	.539**	–					
5. cvSDNN	− .215**	− .161**	.185**	.928**	–				
6. lnRMSSD	.063	− .636**	.653**	.891**	.750**	–			
7. cvRMSSD	.051	− .409**	.428**	.875**	.830**	.964**	–		
8. lnHF	− .067	− .523**	.539**	.843**	.745**	.924**	.913**	–	
9. cvHF	− .099*	− .262**	.279**	.781**	.787**	.834**	.897**	.959**	–

B: Women	1	2	3	4	5	6	7	8	9
1. HFhz	–								
2. HR	− .044	–							
3. IBI	.040	− .999**	–						
4. lnSDNN	− .161**	− .555**	.572**	–					
5. cvSDNN	− .211**	− .194**	.214**	.924**	–				
6. lnRMSSD	.060	− .698**	.711**	.891**	.729**	–			
7. cvRMSSD	.059	− .483**	.499**	.882**	.817**	.964**	–		
8. lnHF	− .083	− .571**	.584**	.865**	.758**	.935**	.932**	–	
9. cvHF	− .111*	− .311**	.326**	.805**	.806**	.837**	.908**	.958**	–

C: Men	1	2	3	4	5	6	7	8	9
1. HFhz	–								
2. HR	− .172**	–							
3. IBI	.165**	− .999**	–						
4. lnSDNN	− .109	− .446**	.472**	–					
5. cvSDNN	− .190**	− .091	.121*	.932**	–				
6. lnRMSSD	.087	− .560**	.581**	.900**	.774**	–			
7. cvRMSSD	.049	− .329**	.353**	.886**	.852**	.967**	–		
8. lnHF	− .058	− .483**	.502**	.845**	.745**	.916**	.895**	–	
9. cvHF	− .116*	− .232**	.254**	.800**	.796**	.846**	.893**	.964**	–

In Table, A represents correlations between variables of interest for the full sample (n = 628). B and C represent these correlations split by women and men, respectfully. Respiration is indexed using HF peak (HFhz) values; HR (heart rate) is in beats per minute; IBI (inter-beat-intervals) are in milliseconds; SDNN represents the standard deviation of inter-beat-intervals (ms); RMSSD reflects the root mean square of successive differences (ms); HF represents high-frequency power (ms^2^); (ln) represents natural log-transformed values; cv represents coefficient of variation (significant *p*-values bolded)

*p < .05 **p < .01

**Fig. 2:**
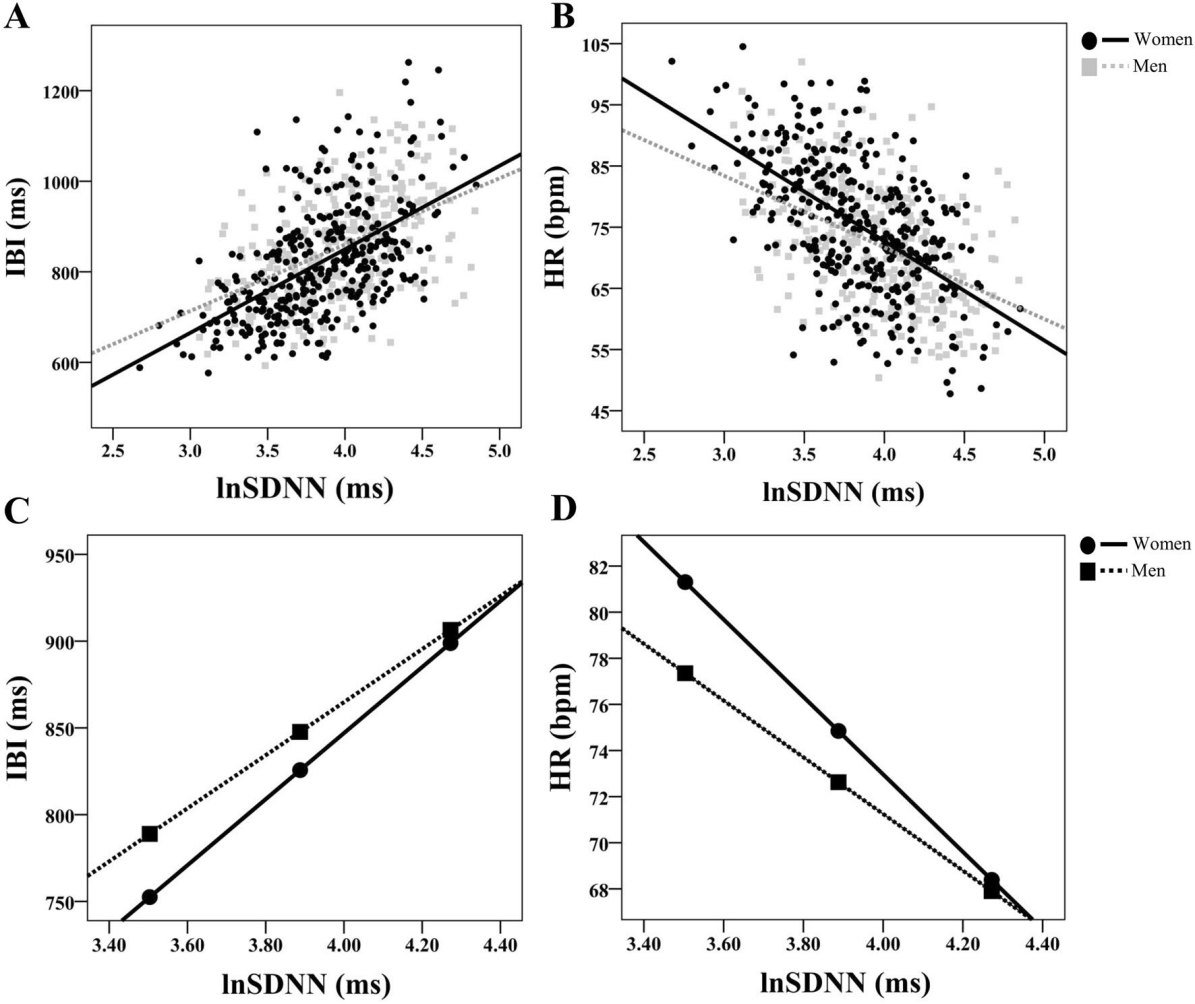
Gender differences in the association between SDNN and cardiac chronotropy. **A** and **B** Show scatterplots of natural log-transformed (ln) standard deviation of inter-beat-intervals (lnSDNN) and both inter-beat-intervals (IBI in ms) and heart rate (HR in beats per minute; bpm), respectively. Within these plots, slopes are labeled differentially as a function of gender. **C** and **D** Depict conditional effects of significant moderation. Higher and lower estimates of SDNN were derived from ± 1SD from the mean (see “Methods” for details)

**Fig. 3:**
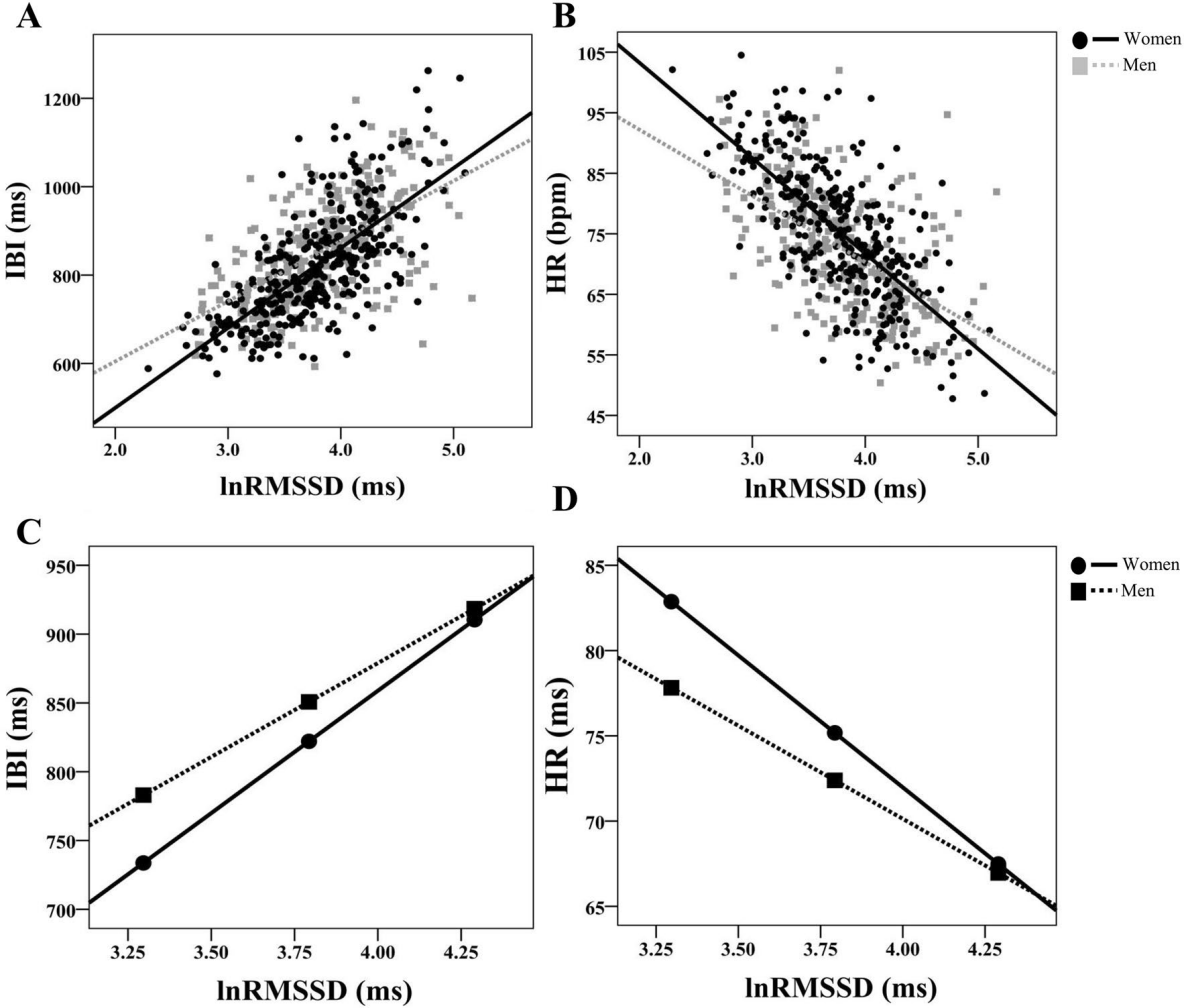
Gender differences in the association between RMSSD and cardiac chronotropy. **3** and **B** Show scatterplots of natural log-transformed (ln) root mean square of successive differences (lnRMSSD) and both inter-beat-intervals (IBI in ms) and heart rate (HR in beats per minute; bpm), respectively. Within these plots, slopes are labeled differentially as a function of gender. **C** and **D ** depict conditional effects of significant moderation. Higher and lower estimates of RMSSD were derived from ± 1SD from the mean (see “Methods” for details)

**Fig. 4:**
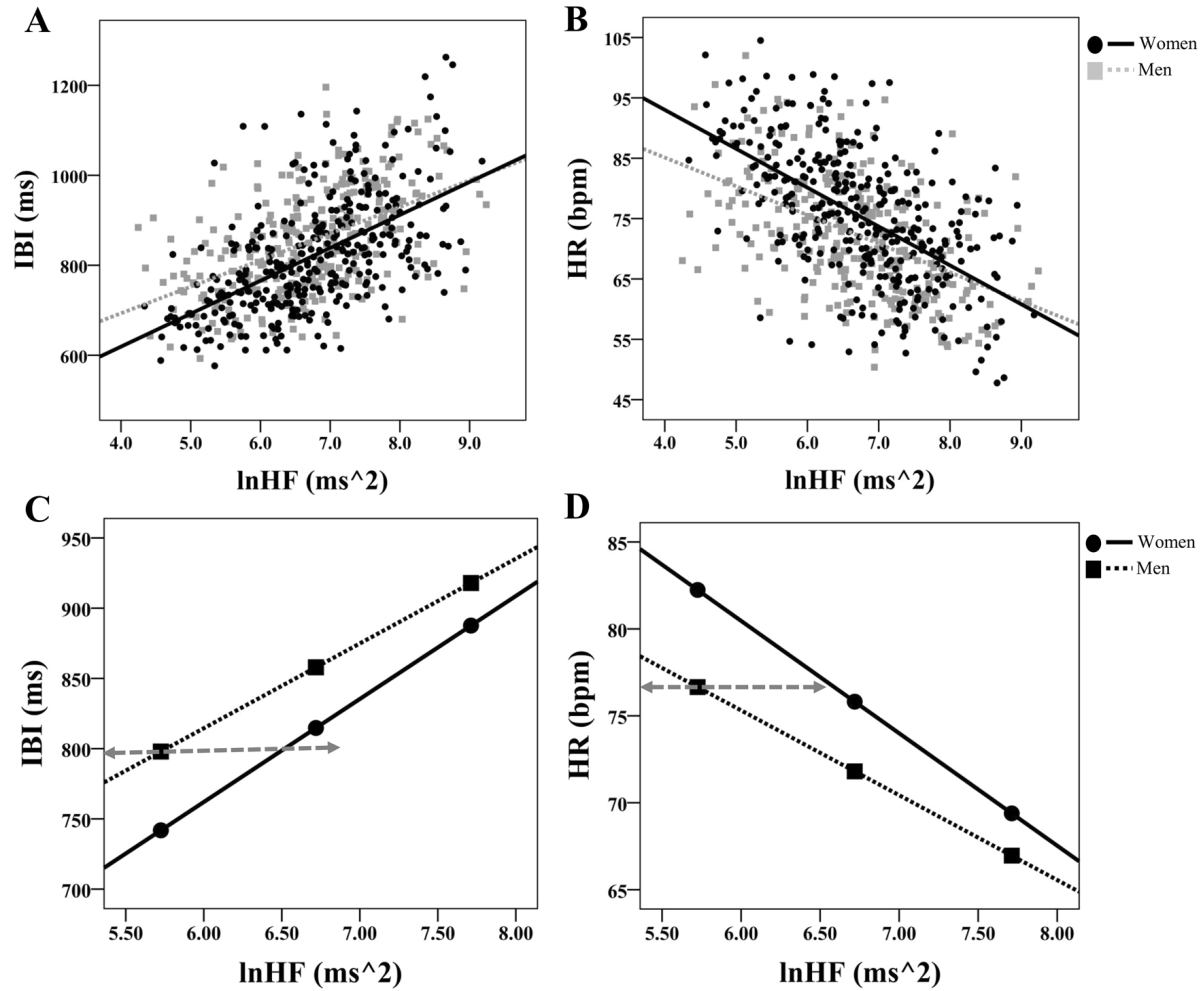
Gender differences in the association between HF and cardiac chronotropy. **A** and **B** Show scatterplots of natural log-transformed (ln) high-frequency (lnHF) and both inter-beat-intervals (IBI in ms) and heart rate (HR in beats per minute; bpm), respectively. Within these plots, slopes are labeled differentially as a function of gender. **C** and **D** Depict conditional effects of significant moderation. Importantly, at any given level of HRV, the heart period is quicker in women relative to men (see **C** for example). For any given level of HR, HRV is higher in women relative to men (see **D** for example). For example, an IBI of ~ 800 ms (**C**) and an HR of ~ 77 bpm (**D**) yields a natural log-transformed HF-HRV value of ~ 6.50 for women and ~ 5.75 for men (see horizontal lines). Higher and lower estimates of HF were derived from ± 1SD from the mean (see “Methods” for details)

Women showed stronger associations between IBI and lnRMSSD (z = 2.80, *p* = 0.005; Fig. 3A) and cvRMSSD (z = 2.23, *p* = 0.026) compared to men. No other significant differences between men and women were observed between measures of HRV and heart period. Similarly, women and men both show significant associations between heart rate and HRV measures. However, women showed significantly stronger associations between HR and both lnRMSSD (z = − 2.87, *p* = 0.004; Fig. 3B) and cvRMSSD (z = − 2.30, *p* = 0.020), but not lnSDNN (z = − 1.81, *p* = 0.070; Fig. 2B), cvSDNN (z = − 1.31, *p* = 0.190), lnHF (z = − 1.52, *p* = 0.129; Fig. 4B), and cvHF (z = − 1.06, *p* = 0.289) compared to men. There was a significant negative association between HF_peak_ and lnSDNN (r = − 0.158, *p* < 0.001), cvSDNN (r = − 0.215, *p* < 0.001), and cvHF (r = − 0.099, *p* = 0.013). These patterns of results were evident in both men and women. However, lnHF, lnRMSSD, and cvRMSSD were not significantly associated with HF_peak_ in the total sample and women and men independently; correlation coefficients did not significantly differ between men and women in this regard. All correlation coefficients for variables of interest stratified by gender are presented in Table 2B and C.

### Moderation Analyses

All moderation tests included BMI and respiration (HF_peak_) as covariates. Gender significantly moderated the association between SDNN and IBI (R^2^_Δ_ = 0.004, B = 0.051 (0.026), [0.001, 0.102], r_*partial*_ = 0.080, *p* = 0.044; Fig. 2C) such that women (B = 0.232 (0.017), [0.198, 0.266], r_*partial*_ = 0.472, *p* < 0.001) showed a stronger association compared to men (B = 0.18 (0.019), [0.143, 0.217], r_*partial*_ = 0.357, *p* < 0.001). Gender also moderated the association between SDNN and HR (R^2^_Δ_ = 0.005, B = − 0.054 (0.026), [− 0.105, − 0.004], r_*partial*_ = 0.084, *p* = 0.036; Fig. 2D), such that women also showed a stronger negative association (B = − 0.224 (0.018), [− 0.259, − 0.190], r_*partial*_ = − 0.456, *p* < 0.001) compared to men (B = − 0.170 (0.019), [-0.207, -0.133], r_*partial*_ = − 0.335, *p* < 0.001). The Johnson-Neyman technique showed that in individuals with natural-log transformed SDNN under approximately 4.2, women showed shorter IBI and higher HR compared to men (each *p* < 0.05). Gender did not significantly moderate the association between cvSDNN and IBI or HR (each *p* > 0.167).

Gender significantly moderated the association between RMSSD and IBI (R^2^_Δ_ = 0.009, B = 0.055 (0.018), [0.049, 0.160], r_*partial*_ = 0.125, *p* = 0.002; Fig. 3C) such that women (B = 0.214 (0.021), [0.190, 0.238], r_*partial*_ = 0.579, *p* < 0.001) showed a stronger positive association compared to men (B = 0.159 (0.013), [0.134, 0.184], r_*partial*_ = 0.447, *p* < 0.001). Gender also moderated the association between RMSSD and HR (R^2^_Δ_ = 0.009, B = − 0.057 (0.018), [− 0.092, − 0.022], r_*partial*_ = − 0.126, *p* = 0.002; Fig. 3D), such that women (B = − 0.209 (0.012), [− 0.233, − 0.185], r_*partial*_ = − 0.565, *p* < 0.001) showed a stronger negative association compared to men (B = − 0.153 (0.013), [− 0.178, − 0.127], r_*partial*_ = − 0.427, *p* < 0.001). The Johnson-Neyman technique showed that in individuals with natural-log transformed RMSSD under approximately 4.06, women showed shorter IBI and higher HR compared to men (each *p* < 0.05). Gender also moderated the associations between cvRMSSD and both IBI (R^2^_Δ_ = 0.010, B = 0.071 (0.025), [0.022, 0.120], r_*partial*_ = 0.113, *p* = 0.005) and HR (R^2^_Δ_ = 0.011, B = − 0.072 (0.025), [− 0.123, − 0.023], r_*partial*_ = − 0.113, *p* = 0.004). Women showed a significantly stronger associations (IBI: B = 0.183 (0.018), [0.148, 0.218], r_*partial*_ = 0.383, *p* < 0.001; HR: B = − 0.176 (0.019), [− 0.211, − 0.141], r_*partial*_ = − 0.369, *p* < 0.001) compared to men (IBI: B = 0.112 (0.018), [0.078, 0.147], r_*partial*_ = 0.250, *p* < 0.001; HR: B = − 0.104 (0.018), [− 0.139, − 0.070], r_*partial*_ = − 0.231, *p* < 0.001) however associations are attenuated in each gender compared to unadjusted RMSSD associations. The Johnson-Neyman technique showed that in individuals with natural log-transformed cvRMSSD under approximately 1.96, women showed shorter IBI and higher HR compared to men (each *p* < 0.05).

On the cusp of statistical significance, gender moderated the association between HF and IBI (R^2^_Δ_ = 0.004, B = 0.018 (0.009), [− 0.008, 0.037], r_*partial*_ = 0.080, *p* = 0.060; Fig. 4C) such that women (B = 0.090 (0.007), [0.076, 0.102], r_*partial*_ = 0.475, *p* < 0.001) showed a stronger positive association compared to men (B = 0.071 (0.007), [0.057, 0.085], r_*partial*_ = 0.373, *p* < 0.001). Gender also moderated the association between HF and HR (R^2^_Δ_ = 0.004, B = − 0.019 (0.010), [− 0.038, 0.002], r_*partial*_ = − 0.080, *p* = 0.053; Fig. 4D) such that women (B = − 0.087 (0.007), [− 0.100, − 0.074], r_*partial*_ = − 0.463, *p* < 0.001) showed a stronger negative association compared to men (B = − 0.068 (0.007), [− 0.082, − 0.054], r_*partial*_ = − 0.357, *p* < 0.001). The Johnson-Neyman technique showed that in individuals with natural-log transformed HF under approximately 7.9, women showed shorter IBI and higher HR compared to men (each *p* < 0.05). Gender did not moderate the associations between cvHF and neither IBI nor HR (each *p* > 0.181).

No moderation models depicted in Fig. 1C (IBI /HR as the independent variable, gender as the moderator, and both unadjusted and adjusted HRV measures as the dependent variables) yielded statistical significance (each *p* > 0.254).

## Discussion

The current study is the first investigation to consider the impact gender may have on the association between HRV and cardiac chronotropy. Our novel results showed that, controlling for body mass index and respiration, gender indeed moderated the association between indices of HRV and indices of cardiac chronotropy. Specifically, women showed significantly stronger associations between unadjusted HRV measures (SDNN, RMSSD, HF) and both IBI and HR. Overall, these results highlight core gender differences in chronotropic control of the heart such that parasympathetic activity, as indexed by HRV, is of greater impact on cardiac chronotropy (i.e., HR and IBI) in women compared to men.

Johnson-Neyman results suggest that at lower levels of HRV, women showed significantly higher HR and shorter IBI relative to men, however this gap narrowed with higher HRV. Importantly, at any given level of HRV, the heart period is quicker in women relative to men (see Fig. 4C). For any given level of HR, HRV is higher in women relative to men (see Fig. 4D). For example, an IBI of ~ 800 ms (Fig. 4C) and an HR of ~ 77 bpm (Fig. 4D) yields a natural log-transformed HF-HRV value of ~ 6.50 for women and ~ 5.75 for men (see horizontal lines in Fig. 4C and D). Therefore, these data are in direct line with epidemiological studies and a prior meta-analysis (Koenig & Thayer, 2016), and illustrate women tend to have higher HR and lower IBI, yet at any given level show higher HRV relative to men.

### Implications for Heart Rate Variability Research

It is imperative that where possible, researchers work to understand how women and men may differ in the association between HRV and psychophysiological outcomes of interest. Results may yield interactions similar to those presented in the current study, or such gender differences may fundamentally change hypotheses and results (e.g., Williams et al., 2018; Spangler et al., 2021). For example, we showed gender to moderate the association between resting HRV and difficulties in emotion regulation, such that women showed a stronger association compared to men (Williams et al., 2018). Overall, at the very least and where adequately powered, researchers should stratify their samples by gender to ensure that observed results are in the same and/or hypothesized direction in both women and men. Such recommendations comport with the strong NIH recommendations to report results stratified by gender (Clayton & Collins, 2014; Clayton, 2016; Ramirez et al., 2017).

As previously mentioned, ACh slows depolarization (the heart period) and increases heart period variability, making these two indices of heart rhythm “hardwired” as it were (see de Geus et al., 2019, for review). On the issue of HRV adjustments, one underlying assumption is that the association between HRV and the resulting heart period is relatively equal across individuals. Evinced by our data, the association between HRV and heart period is not equal across women and men. In line with the current data, another study showed higher HF modulation of HR in women compared to men (Huikuri et al., 1996). Importantly, converging evidence suggests greater ACh *sensitivity* in females compared to males. One study showed that greater activation of the vagus nerve led to greater pre-and post-synaptic cardiac ACh effects in female rats relative to male rats, thereby providing direct evidence of greater cardiac ACh effects in females relative to males (Du et al., 1994). Another study showed acetylcholinesterase activity, or the breakdown of ACh, is higher in newborn males compared to females, suggesting that ACh breakdown occurs slower in females compared to males as early as birth in rats (Loy & Sheldon, 1987). One review article (Dart et al., 2002) has detailed extensive research outlining gender differences in autonomic control of the heart, and specifically outlines converging evidence that sex hormones such as estrogen, are primary mechanisms in mediating ACh sensitivity in women. Importantly, this review article outlines evidence showing that the effect of sex hormones on the synthesis and clearance of neurotransmitters, including ACh, occur in both the heart and vasculature (Dart et al., 2002). If ACh is the primary neurotransmitter that increases vagally-mediated HRV and the heart period, and women show greater sensitivity to ACh, then the association between HRV and cardiac chronotropy (i.e., IBI) *should indeed* be stronger in women compared to men. Therefore, prior research regarding gender differences in ACh sensitivity (e.g., Loy & Sheldon, 1987; Taddei et al., 1996; Du et al., 1994; Dart et al., 2002), and more generally autonomic control of the heart (Huikuri et al., 1996; Dart et al., 2002), provide direct and compelling physiological evidence for our current findings. Taken together, prior work and the current data highlight problems with adjusting HRV for the heart period; this “hardwired” association is not consistent across men and women.

With regard to adjustments in HRV for the heart period, a proposed physiological argument outlines that vagal activity “independently” influences both HRV and the heart period, and supposedly the heart period impacts HRV, but not vice versa (de Geus et al., 2019). In contrast, we theorize that vagal outflow, as indexed by HRV, should influence heart period and not the reverse. The data presented here support our perspective, as gender *did not* moderate the association between cardiac chronotropy and HRV (Fig. 1C); instead, only when HRV was the independent variable did gender moderate this association (Fig. 1A). As such, from a theoretical standpoint, any correction to HRV using IBI or HR would likely remove variance of interest. In fact, authors agree in that they state, “We specifically caution that adjustment approaches may in fact remove meaningful variance in outcomes of interest that can be attributed to autonomic and neurophysiological phenomena” (de Geus et al., 2019, p. 21). This is further supported by the current findings in that the associations between HRV and cardiac chronotropy are attenuated when the “adjusted” HRV indices are used; the magnitude of this attenuation is a measure of the extent of this shared variance. Furthermore, a recent mega-analysis showed cortical thickness in brain regions associated with vagal outflow to be significantly associated with HRV, but *not* HR (Koenig et al., 2020). Such regions of interest included left and right caudal anterior cingulate cortex, left and right insula, and the left lateral orbitofrontal cortex (Koenig et al., 2020). This is of extreme importance from a neurophysiological perspective; in 1218 humans, cortical thickness in 14 brain regions responsible for vagal outflow was associated with HRV but not HR (Koenig et al., 2020), thereby providing direct and compelling evidence that HRV likely impacts the heart period. Interestingly, this study also showed women to have greater cortical thickness in all brain regions associated with vagal outflow, with exception of left and right caudal anterior cingulate cortex (thinner in females). Therefore, gender differences in cortical thickness might explain sex differences in cardiac function on a neural structural level.

Gender did not moderate the association between cvHRV (cvSDNN and cvHF) and neither IBI nor HR. This is important, as despite a significant decrease in the magnitude of the association between HRV and cardiac chronotropy period using adjusted compared to unadjusted HRV, this decrease in magnitude was not the same across gender. Notably, this was not the case with RMSSD, as gender moderated the association between both RMSSD and cvRMSSD and the heart period, thereby suggesting that the adjustment worked similarly for both women and men. This further suggests that the attenuation of the HRV and heart period association, when applying the cv transformation, is inconsistent across HRV measures (e.g., HF and RMSSD). Taken together, our data suggest that the cv adjustment to HRV is neither consistent across individuals nor consistent across HRV measures. Future research should examine the impact other demographics, such as race, may have on the association between HRV and cardiac chronotropy.

Another interesting finding was that the associations between cvHRV and respiration—indexed using HF_peak_—appear stronger in some cases compared to unadjusted HRV measures. For example, the association between HF and respiration was small and non-significant, whereas the association between cvHF and respiration was small yet significant. SDNN and cvSDNN was significantly associated with respiration, however the cvSDNN association was slightly stronger (not statistically significant yet trending in that direction). Neither RMSSD variable was associated with respiration. Therefore, future research should also continue to investigate how the cv transformation impacts the association between respiration and HRV measures.

In sum, we advocate strongly *against “*routinely adjusting” HRV for the heart period. “If an adjustment for cardiac chronotropic state (heart period or rate) is employed, it is incumbent on the author(s) to justify the specific adjustment within a given context” (de Geus et al., 2019, p. 21). Thus, one must be cautious and carefully consider their research question and sample demographics prior to deciding to “adjust” HRV variables for heart period; such a decision must occur on a case-by-case basis. Nevertheless, *universal* or *routine* “adjustments” to measures of HRV appear unjustified and problematic at best; continued research is needed to understand the reliability and validity of such adjustments. Here, we highlight that a cvHRV adjustment would be particularly problematic across women and men.

### Limitations and Future Directions

The current study is not without its limitations. As mentioned, estrogen is the likely mechanism underlying greater ACh sensitivity in women compared to men (Taddei et al., 1996; Sarabi et al., 1999; Pinto et al., 1997; Dart et al., 2002). However, women either do not differ or show a lesser difference in estrogen compared to men in older individuals, particularly post-menopause in women. Thus, it would be interesting to explore and to understand if gender moderates the association between HRV and cardiac chronotropy across age groups, as the current sample reflects young and healthy individuals. A second limitation of the study is that only baseline cardiovascular data was available. It would be extremely interesting to consider how gender differences in the association between HRV and cardiac chronotropy may be more or less evident under alternative conditions (orthostatic challenge, psychological tasks, etc.).

## Conclusions

Overall, data from our large sample suggests that the association between HRV and subsequent cardiac chronotropy (IBI and HR) significantly differs between women and men. At a given level of HRV, especially lower levels of HRV, the heart period is quicker in women compared to men; this is important, as these data suggest the difference in HR between men and women vary as a function HRV. Given such findings, we strongly advocate against routinely “adjusting” HRV for heart period, as such transformations are unjustified and problematic at best, especially across gender. We must continue to be both careful and mindful in our HRV measurements, practices, and analyses. Last but not least, it is imperative that scientists consider demographic factors—especially gender—in HRV research.
